# Heterologous Expression of *Alteromonas macleodii* and *Thiocapsa roseopersicina* [NiFe] Hydrogenases in *Synechococcus elongatus*

**DOI:** 10.1371/journal.pone.0020126

**Published:** 2011-05-26

**Authors:** Philip D. Weyman, Walter A. Vargas, Yingkai Tong, Jianping Yu, Pin-Ching Maness, Hamilton O. Smith, Qing Xu

**Affiliations:** 1 Department of Synthetic Biology and Bioenergy, The J. Craig Venter Institute, Rockville, Maryland, United States of America; 2 National Renewable Energy Laboratory, Golden, Colorado, United States of America; Universidad Miguel Hernandez, Spain

## Abstract

Oxygen-tolerant [NiFe] hydrogenases may be used in future photobiological hydrogen production systems once the enzymes can be heterologously expressed in host organisms of interest. To achieve heterologous expression of [NiFe] hydrogenases in cyanobacteria, the two hydrogenase structural genes from *Alteromonas macleodii* Deep ecotype (AltDE), *hynS* and *hynL*, along with the surrounding genes in the gene operon of HynSL were cloned in a vector with an IPTG-inducible promoter and introduced into *Synechococcus elongatus* PCC7942. The hydrogenase protein was expressed at the correct size upon induction with IPTG. The heterologously-expressed HynSL hydrogenase was active when tested by *in vitro* H_2_ evolution assay, indicating the correct assembly of the catalytic center in the cyanobacterial host. Using a similar expression system, the hydrogenase structural genes from *Thiocapsa roseopersicina* (*hynSL*) and the entire set of known accessory genes were transferred to *S. elongatus*. A protein of the correct size was expressed but had no activity. However, when the 11 accessory genes from AltDE were co-expressed with *hynSL*, the *T. roseopersicina* hydrogenase was found to be active by *in vitro* assay. This is the first report of active, heterologously-expressed [NiFe] hydrogenases in cyanobacteria.

## Introduction

Hydrogen (H_2_) production from photosynthetic microorganisms is an attractive strategy to store solar energy as a fuel [1]. H_2_ fuel cells can provide carbon-free power; however, most H_2_ currently in use derives from fossil fuels [1]. Development of photobiological H_2_ production using photosynthetic microorganisms such as cyanobacteria and micro-algae can provide an alternative to fossil fuels by using the energy of the sun to convert H_2_O into H_2_.

H_2_ can be produced by cyanobacteria using either nitrogenase or hydrogenase enzymes [2]. Hydrogenases catalyze the reversible reduction of protons to H_2_ and can be divided into three phylogenetically-distinct categories that correlate with the metal composition of the active site: [FeFe], [NiFe], and the [Fe] hydrogenases of methanogens [3], [4]. Two different groups of [NiFe] hydrogenases, the uptake hydrogenases and the bidirectional hydrogenases, have been found in many cyanobacterial genomes [2]. The uptake hydrogenases in cyanobacteria function largely in recycling H_2_ produced as a byproduct of nitrogen fixation while bidirectional hydrogenases have been implicated in disposing of excess reductant as H_2_ [5], [6].

H_2_ production from photosynthetic microbes such as cyanobacteria requires hydrogenases to be tolerant of oxygen produced from photosynthesis if H_2_ production is to occur during daytime. Of the major categories of hydrogenases, [FeFe] hydrogenases are the most O_2_-sensitive and are irreversibly destroyed by exposure to oxygen [7]. Most [NiFe] hydrogenases are temporarily inactivated by O_2_ but can be reactivated upon returning to anaerobic conditions given sufficient reducing conditions [7]. All cyanobacterial [NiFe] hydrogenases studied thus far are sensitive to O_2_ and function only briefly in aerobic conditions before being inactivated [8]. Nonetheless, several [NiFe] hydrogenases from other microorganisms maintain activity in the presence of oxygen, including those from *Ralstonia eutropha* [9], *Rubrivivax gelatinousous* [10] and *Alteromonas macleodii* [11].

Using O_2_-tolerant hydrogenases in future cyanobacterial hydrogen production systems will require their heterologous expression in cyanobacteria, and expression is currently a barrier to the wide-spread use of foreign hydrogenases in cyanobacteria. The catalytic core of [NiFe] hydrogenases generally consists of two subunits, one large (*ca*. 60 kDa) and one small (*ca*. 30 kDa). The large subunit contains the [NiFe] catalytic site and requires an extensive set of accessory proteins to assemble an active catalytic site [12]. Maturation of the small subunit is not as well understood, but some [NiFe] hydrogenases require specific accessory proteins to assist in this process [13]. The accessory proteins are usually specific for the hydrogenase with which they have co-evolved, but may be active on closely related hydrogenases from another species [14], [15].

The complete and clustered set of accessory genes from AltDE may simplify the task of heterologous expression [16]; however, other organisms, including *Thiocapsa roseopersicina* that contains a related [NiFe] hydrogenase, have accessory genes distributed throughout the genome [17]. Although the *T. roseopersicina* genome has not been sequenced, mutational analysis has identified several accessory genes (*hynD*, *hupCDHIK* and *hypC1C2DEF*) [17], [18], [19]. Here, we report the heterologous expression of active [NiFe] hydrogenases from AltDE and *T. roseopersicina* in the cyanobacterium *Synechococcus elongatus* PCC7942. Heterologous expression of an active [NiFe] hydrogenase has not been reported previously in cyanobacteria, and the development of systems for heterologous expression of hydrogenases in cyanobacteria may open up new possibilities for photobiological hydrogen production.

## Results

### Construction of the *hoxYH* hydrogenase mutant in *S. elongatus* PCC7942

The genome of *S. elongatus* PCC7942 contains the *hoxYH* genes encoding one [NiFe] bidirectional hydrogenase (HoxYH) (http://genome.jgi-psf.org/synel/synel.home.html). To eliminate background hydrogenase activity in *S. elongatus* to better detect the activity from heterologously-expressed enzymes, we knocked out the endogenous hydrogenase by transforming with a plasmid (pPW416) that would replace the *hoxYH* genes with an antibiotic cassette via DNA recombination. Complete segregation of the mutation-containing sequence in *S. elongatus* was confirmed by PCR and southern blot analysis (Figs. S1 and S2). The *S. elongatus* knockout mutant, named PW416, lacked any detectable hydrogenase activity (Table S2).

### Expression of AltDE HynSL hydrogenase in *S. elongatus* PCC7942

**Figure 1:**
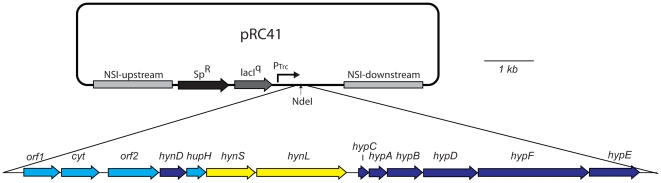
Diagram of pRC41 used for expression of AltDE hydrogenase HynSL in *S. elongatus*. Arrows indicate the direction of open reading frames. The figure was drawn to scale.

AltDE contains one [NiFe] hydrogenase, HynSL, which has been characterized as oxygen-tolerant and thermostable [11]. The structural genes, *hynSL*, are surrounded by 11 genes encoding accessory proteins involved in the assembly and maturation of the [NiFe] hydrogenase catalytic site and the hydrogenase complex. A subset of these genes has been determined to play a critical role in hydrogenase maturation [16]. In order to express HynSL in *S. elongatus*, plasmid pRC41 was constructed by cloning the hydrogenase structural genes, *hynSL*, and the 11 adjacent genes into an expression plasmid [16] (Fig. 1). This plasmid contains a copy of the *lacI* gene [20] and allows for expression of the AltDE hydrogenase operon from the IPTG-inducible P_Trc_ promoter. The plasmid pRC41 also contains flanking sequence for insertion of the hydrogenase gene cluster into “neutral site I” (NSI) of the *S. elongatus* chromosome via homologous DNA recombination [21]. The construct was introduced into the *S. elongatus* hydrogenase knockout strain, PW416, to create strain RC41.

**Figure 2:**
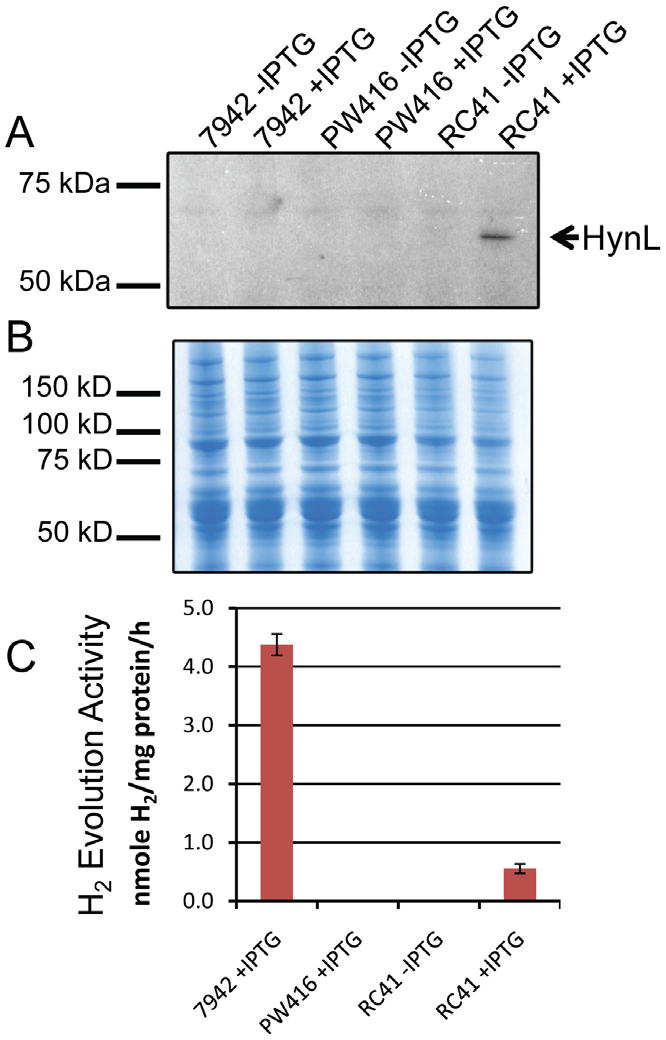
Heterologous expression of AltDE hydrogenase HynSL in *S. elongatus*. **A.** Western blotting of protein extracts from *S. elongatus* cell cultures. Each lane contains 25 µg total proteins from cells treated with or without IPTG. The arrow indicates the position of the HynL band. **B.** Coomassie blue staining of a duplicate protein gel as in Panel A showing equal loading in each lane. **C.** Determination of the hydrogenase activity of the heterologously expressed HynSL. *In vitro* hydrogen evolution assays were performed on protein samples described in Panel A. Two controls, 7942-IPTG and PW416-IPTG, were not included since their activity levels are same as those from 7942+IPTG and PW416+IPTG, respectively. Error bars indicate standard deviations of the mean from at least three samples. The experiment was repeated at least three times with similar results.

To examine the expression of HynSL, RC41 cultures expressing the AltDE hydrogenase gene operon were grown and induced with IPTG, and protein extracts from lysed cells were separated on an SDS-PAGE gel for immunoblotting. Western blotting was performed with antiserum raised against the *T. roseopersicina* [NiFe] hydrogenase large subunit, HynL. When induced with IPTG, a single band corresponding to the mature form of the AltDE HynL (67 kDa) was detected (Fig. 2A). Without IPTG induction, no HynL band was detected. As a control, a duplicate gel was stained with Coomassie blue to confirm equal loading in each lane (Fig. 2B). IPTG was added to a final concentration of 5, 20, 100, or 200 µM to RC41 cultures to determine the optimal concentration of IPTG for protein expression. Western blotting analysis indicated that 100 µM IPTG yielded maximal expression of HynL (data not shown) and this concentration of IPTG was used for all future experiments.

To determine whether the AltDE HynSL protein was expressed with an active catalytic site in *S. elongatus* PCC7942, *in vitro* H_2_ evolution assays were performed with protein extracts from RC41. As expected, no hydrogenase activity was detected in the PW416 (Δ*hoxYH*) strain (Fig. 2C). Hydrogenase activity was detected in strain RC41 expressing both hydrogenase and accessory proteins from AltDE, and activity was strongly induced by IPTG. The activity from the heterologously expressed hydrogenase in RC41 represented one tenth of the native activity in the wild type *S. elongatus* strain (Fig. 2C). A small amount of activity was detected in the absence of IPTG induction, indicating slightly leaky expression from the P_Trc_ promoter.

### Expression of *T. roseopersicina* HynSL hydrogenase in *S. elongatus* PCC7942

**Figure 3:**
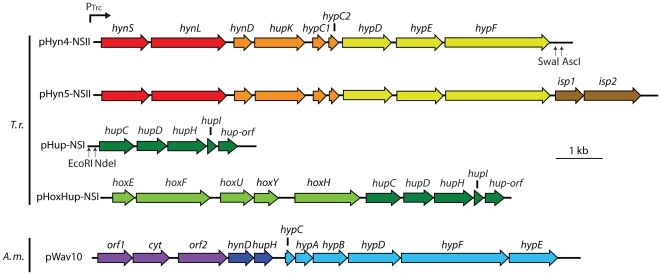
Diagram of constructs used in heterologous expression of *T. roseopersicina* HynSL in *S. elongatus*. Arrows indicate the direction of open reading frames. The figure was drawn to scale.

In addition to expressing AltDE HynSL, we expressed its related stable hydrogenase, HynSL, from *T. roseopersicina* in *S. elongatus*. We assembled the sequences of *T. roseopersicina* accessory genes into two sets of plasmids. In the first set of plasmids, pHyn4-NSII, *hynD* (encoding the protease) as well as *hupK* and *hypC1C2DEF* were assembled along with the hydrogenase structural genes *hynSL* in an IPTG-inducible vector (Fig. 3), which could integrate into the “neutral site II” (NSII) of *S. elongatus* through homologous recombination [21]. The plasmid was introduced into the hydrogenase knockout mutant (PW416) to make strain Hyn4. We further modified plasmid pHyn4-NSII to include two additional genes, *isp1* and *isp2*. These two genes are found between *hynS* and *hynL* in the *T. roseopersicina* chromosome [22]. They were removed in the construction of pHyn4-NSII but were added back downstream of the *hypDEF* genes in the construction of pHyn5-NSII (Fig. 3). *Isp1* and *Isp2* are not predicted to play a role in hydrogenase maturation but rather confer electron transfer necessary for *in vivo* hydrogenase activity [23]. Plasmid pHyn5 was mobilized into the hydrogenase knockout strain to make Hyn5.

In the second set of plasmids, pHup-NSI, the *T. roseopersicina* accessory genes *hupCDHI* and an ORF of unknown function were assembled in an IPTG-inducible vector, which could recombine at the *S. elongatus* NSI locus (Fig. 3). After mobilization of pHup-NSI into the hydrogenase knockout mutant, PW416, the resulting strain was named Hup. The pHup-NSI plasmid was also mobilized into Hyn4 and Hyn5 to create strains Hyn4/Hup and Hyn5/Hup, respectively.

**Figure 4:**
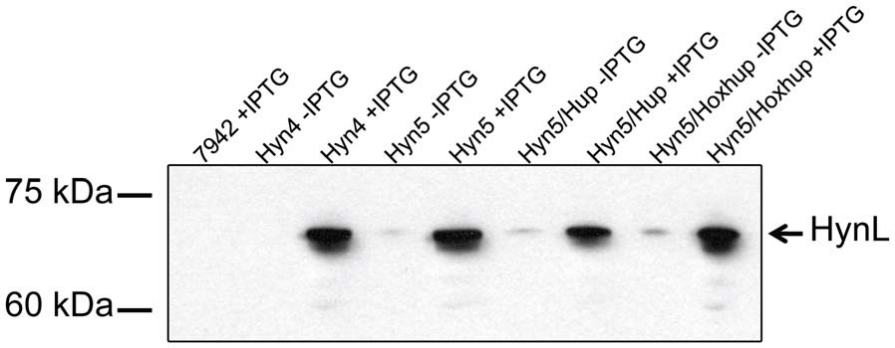
Detection of the *T. roseopersicina* hydrogenase large subunit HynL in *S. elongatus*. Western blotting was performed on protein extracts from *S. elongatus* strains (wild-type PCC7942, Hyn4, Hyn5, Hyn5/Hup, and Hyn5/Hoxhup) by using anti-HynL antisera. Hyn4/Hup was not included since the expression level in this strain was similar to that in Hyn4. Each lane contains 25 µg total proteins from cells treated with or without IPTG. Strain Hyn4 carries the *T. roseopersicina* genes *hynSL*, *hynD*, *hupK*, and *hypC_1_C_2_DEF* while strain Hyn5 carries the genes from Hyn4 as well as *isp1* and *isp2*. Strain Hyn5/Hup contains the *T. roseopersicina* genes *hupCDHIorf* and the genes from Hyn5. Strain Hyn5/Hoxhup contains the *T. roseopersicina* genes *hoxEFUYH* in addition to the genes from Hyn5/Hup.

*S. elongatus* cell cultures expressing different combinations of *T. roseopersicina* structural and accessory genes were induced with IPTG, and protein extracts prepared from lysed cells were analyzed by immunoblot after SDS-PAGE electrophoresis using anti-HynL antisera. A band corresponding to the correct size of the mature form of HynL was detected (62 kDa), and increased expression was observed in the presence of IPTG (Fig. 4). Similar levels of HynL expression were observed for all strains that contained HynSL (Fig. 4). *In vitro* hydrogen evolution assays were performed to determine whether the expressed hydrogenase possessed activity. No activity was detected in strains Hyn4, Hyn5, Hyn4/Hup or Hyn5/Hup (data not shown).

### Expression of the *T. roseopersicina* HoxYH in *S. elongatus* PCC7942

Since the *T. roseopersicina* HynSL hydrogenase was not properly assembled into its active form in *S. elongatus* using the native cyanobacterial accessory proteins, we sought to determine whether an enzyme with greater similarity to the cyanobacterial hydrogenase such as HoxYH might be successfully assembled and possess activity when heterologously expressed. *T. roseopersicina* also contains a bidirectional hydrogenase encoded by genes *hoxYH*, which has been shown to interact with the diaphorase subunit encoded by genes *hoxEFU* [23]. The small and large subunits are 44 and 54 percent similar, respectively, to the native bidirectional hydrogenase in *S. elongatus* [24]. Given the similarities between the two proteins, a functional *T. roseopersicina* HoxYH may be able to be assembled and processed by the native *S. elongatus* accessory proteins. To express the *T. roseopersicina* HoxYH hydrogenase in *S. elongatus*, the *hoxEFUYH* gene cluster was cloned upstream of the *hupCDHIorf* genes, resulting in plasmid pHoxhup-NSI (Fig. 3). pHoxhup-NSI with the combined gene cluster was transformed into *S. elongatus* PW416 and Hyn5, to make strains Hoxhup and Hyn5/Hoxhup, respectively. The *S. elongatus* cultures were induced with IPTG, and protein extracts from lysed cells were used for immunoblot analysis. A 62 kDa protein band corresponding to the correct size of the mature form of HynL was observed (Fig. 4). *in vitro* hydrogen evolution assay was performed to determine if an active hydrogenase was produced. No activity was detected for Hoxhup or Hyn5/Hoxhup that contains additional *T. roseopersicina* accessory genes *hynDhupKhypC1C2DEF* (data not shown). Thus, maturation of a functional HoxYH from *T. roseopersicina* requires additional accessory genes that are not able to be complemented by genes in the cyanobacterial host.

### Co-expression of *T. roseopersicina* HynSL and AltDE accessory proteins in *S. elongatus* PCC7942

**Figure 5:**
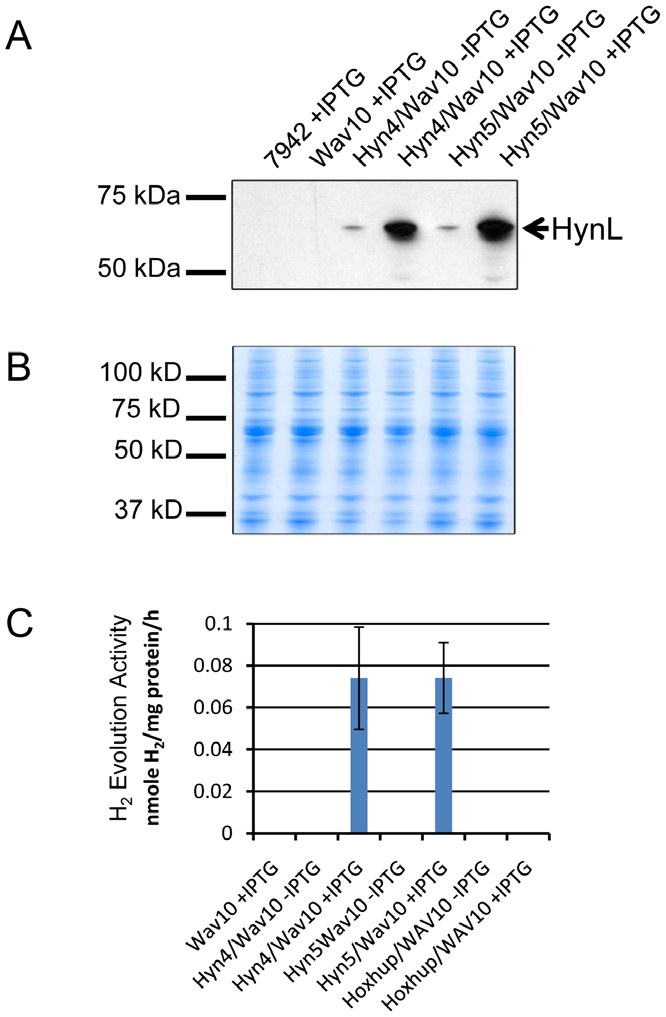
Expression of *T. roseopersicina* hydrogenase with accessory proteins from AltDE in *S. elongatus*. **A.** Detection of the *T. roseopersicina* hydrogenase large subunit HynL in *S. elongatus*. Western blotting was performed with protein extracts of *S. elongatus* strains, wild-type PCC7942, Wav10, Hyn4/Wav10, and Hyn5/Wav10. Each lane contains 25 µg total proteins from samples treated with or without IPTG. **B.** Coomassie blue staining of a duplicate protein gel as in panel A showing equal loading in each lane. **C.** Determination of the hydrogenase activity of the heterologously expressed HynSL. *In vitro* hydrogen evolution assays were performed on protein samples collected from *S. elongatus* strains Wav10, Hyn4/Wav10, Hyn5/Wav10, and HoxHup/Wav10 after treatment with or without IPTG induction. Wav10-IPTG was not included since the activity in this negative control was not affected by IPTG induction. Error bars indicate standard deviations of the mean from at least three samples. The experiment was repeated at least three times with similar results.

Since HynSL from AltDE was active when expressed in *S. elongatus* RC41, we tried to co-express the *T. roseopersicina* HynSL hydrogenase with accessory proteins from AltDE. Plasmid pWAV10 (Fig. 3) was constructed to carry only the 11 genes encoding AltDE accessory proteins and was introduced into the NSI locus of *S. elongatus* strains Hyn4 and Hyn5 described above, creating Hyn4/Wav10 and Hyn5/Wav10, respectively. To serve as a control, pWAV10 was also introduced into the *S. elongatus* hydrogenase knockout strain, PW416, creating Wav10 that contained all AltDE accessory genes, but no structural genes. Expression of the HynL protein in these strains was verified by SDS-PAGE and Western blotting (Fig. 5A). A duplicate gel was stained with Coomassie blue to confirm equal loading in each lane as a loading control (Fig. 5B). *In vitro* H_2_ evolution assays were performed to determine whether the heterologously expressed *T. roseopersicina* HynSL hydrogenase was active when expressed in the cyanobacterium with the AltDE accessory proteins. Hydrogen evolution activity was detected in both Hyn4/Wav10 and Hyn5/Wav10 strains at similar levels in both strains (Fig. 5C). Activity from *T. roseopersicina* HynSL was approximately one tenth of the activity of AltDE HynSL (Figs. 2C and 5C). To determine if the AltDE accessory proteins were capable of producing an active *T. roseopersicina* HoxYH, the plasmid pWav10 was introduced into the *S. elongatus* strain Hoxhup. The resulting strain, Hoxhup/Wav10, did not produce any H_2_ during *in vitro* hydrogen evolution assays after IPTG induction (Fig. 5C).

## Discussion

We have expressed the [NiFe] hydrogenases from AltDE and *T. roseopersicina* in the heterologous host, *S. elongatus* PCC7942. [NiFe] hydrogenases have been previously expressed in heterologous bacterial hosts [14], [15], [25], [26], [27], [28], but to our knowledge this is the first [NiFe] hydrogenase to be heterologously expressed in a cyanobacterium with a fully assembled active site. The ability to heterologously express properly assembled [NiFe] hydrogenases in cyanobacteria has been a technical barrier hindering widespread biotechnological application of hydrogenases with unique properties. In direct photobiological hydrogen production systems, electrons derived from water oxidation through oxygenic photosynthesis are used directly for hydrogen production without being stored as a fixed-carbon intermediate [29]. Development of such a system requires either low partial pressures of O_2_ as has been achieved for brief periods with eukaryotic green algae [30], or the use of an O_2_-tolerant hydrogenase. We have taken a step toward the latter approach by expressing a functional O_2_-tolerant [NiFe] hydrogenase in cyanobacteria. While the AltDE hydrogenase we used is only partially tolerant of up to 1–3 percent O_2_, their expression will enable further progress on photobiological hydrogen production using the many genetic tools available for cyanobacteria [31].

The set of AltDE accessory proteins that was found to be required for maximal activity of AltDE HynSL when heterologously expressed in *Escherichia coli* included HypCABDFE, the protease HynD, HupH, and a protein of unknown function, Orf2 [16]. The *S. elongatus* strain RC41 expressed the above set of accessory proteins along with Orf1 and Cyt. The AltDE proteins Orf1 and Cyt were not found to be necessary for maximal activity when HynSL was heterologously expressed in *E. coli*, but it is unknown whether these proteins affect the maturation efficiency of HynSL in *S. elongatus* PCC7942. The set of AltDE accessory proteins contained in Wav10 was the same as those found on RC41 and was also sufficient to allow for expression of a functional *T. roseopersicina* hydrogenase HynSL in *S. elongatus*. When expressed in *S. elongatus*, AltDE HynSL activity was higher than *T. roseopersicina* HynSL activity, and this may reflect lower levels of activity of the AltDE accessory proteins when acting on the *T. roseopersicina* HynSL. Similarly, lower levels of activity were detected when HynSL was expressed with the AltDE accessory proteins in *E. coli* compared to expression of AltDE HynSL [16].

We assembled the genes encoding the entire known set of *T. roseopersicina* accessory proteins into an artificial operon driven by the P_Trc_ promoter. This set of accessory genes was not sufficient to produce an active HynSL hydrogenase. The *T. roseopersicina* operon included many genes that were also included in the AltDE cluster such as *hypCDEF*, *hynD*, and *hupH*. HupH from AltDE was required for maximal assembly and maturation of AltDE HynSL in *E. coli* [16] and was also identified in *T. roseopersicina*; however, the HupH sequences from AltDE and *T. roseopersicina* share only 11% similarity. It is unknown whether the two HupH proteins function similarly in their respective hosts. Absent from the group of known *T. roseopersicina* accessory genes is *hypAB*. These genes encode proteins that belong to the set of accessory proteins (HypABCDEF) that is found in all species containing [NiFe] hydrogenases [12]. HypAB function to add the Ni atom to the nascent [NiFe] catalytic site. In some systems, the absence of HypAB has been complemented by high concentrations of nickel, but these genes are required for maximal hydrogenase activity [32]. When the AltDE *hypAB* genes were co-expressed with in *T. roseopersicina hynSL*, *hynD*, *hypC1C2DEF* in *E. coli*, no hydrogenase activity was detected [16]. This suggests that the AltDE HypAB proteins alone cannot effectively interact with the rest of the *T. roseopersicina* accessory proteins and that the additional proteins (HupH or Orf2) encoded by Wav10 are important to the maturation of the *T. roseopersicina* hydrogenase.

We also attempted to express the bidirectional hydrogenase from *T. roseopersicina*, HoxYH, in *S. elongatus.* Even after co-expression with the entire set of known *T. roseopersicina* accessory proteins or with the accessory proteins from AltDE, no hydrogenase activity could be detected from strains expressing HoxYH. Missing from the accessory genes known to contribute to maturation of HoxYH is the endo-peptidase, HoxW. This peptidase activity is apparently not able to be complemented by the native *S. elongatus* HoxW that was purposely left intact when constructing the *hoxYH* mutant strain (PW416). This finding is consistent with a previous report demonstrating that HoxW cannot be complemented by other proteases for processing *T. roseopersicina* HoxYH [33].

Heterologous expression of NiFe hydrogenases with fully assembled active sites in cyanobacteria will enable further study of hydrogenase expression in photosynthetic prokaryotic hosts such as cyanobacteria. Further studies are in need to increase expression, improve oxygen tolerance to the atmospheric levels, and establish coupling of the heterologous hydrogenase to the photosynthetic electron transport chain. The successful outcomes will lead to cyanobacterial strains capable of producing hydrogen simultaneously during photosynthesis.

## Materials and Methods

### Strains and growth conditions

Molecular biology techniques were performed according to [34]. *E. coli* cultures were grown at 30°C or 37°C in Luria-Bertaini (LB) broth, or on LB agar plates supplemented with antibiotics as needed (spectinomycin, 50 µg ml^−1^, kanamycin, 25 µg ml^−1^, chloramphenicol, 25 µg ml^−1^). Cyanobacteria were grown in BG11 liquid media [35] or on BG11 agar plates supplemented with antibiotics as needed (spectinomycin, 10 µg ml^−1^; kanamycin, 10 µg ml^−1^; erythromycin, 5 µg ml^−1^). Cells were grown under continuous illumination (40 µE m^−2^ s^−1^) at 28°C in 100 ml cultures with constant shaking or in 500 ml cultures with constant stirring and aeration. Cultures were induced with Isopropyl β-D-1-thiogalactopyranoside (IPTG) at a final concentration of 100 µM and NiCl_2_ at a final concentration of 0.5 µM for 24 hours before the cultures were used for experiments. Cultures of AltDE and *T. roseopersicina* were grown as previously described [11].

### Plasmid construction and genetic manipulation of cyanobacteria

Plasmid pPW416 was constructed to knockout the hydrogenase structural genes *hoxYH* in *S. elongatus* PCC7942 [36]. This vector was designed to leave the upstream *hoxU* and downstream *hoxW* intact (Table 1). To make pPW416, a four piece ligation was performed using the following DNA pieces: 1) A PCR product containing resistance genes to erythromycin (Em^R^) and chloramphenicol (Cm^R^) amplified from pRL2948a using primers EmCm-F and EmCm-R (Table 2) and digested with XhoI and SpeI, 2) A PCR product containing 1-kb of sequence upstream of *S. elongatus hoxY* amplified using primers Hox11 and Hox12 and digested with HindIII and XhoI, 3) a PCR product containing 1-kb of sequence downstream of *hoxH* amplified with primers Hox15 and Hox16 and digested with SpeI and XbaI, and 4) pUC19 digested with HindIII and XbaI. After transformation into *E. coli*, the resulting plasmid was subsequently digested with XbaI and ligated with an XbaI-digested DNA fragment from pRL448 containing a kanamycin resistance gene (Km^R^). The resulting plasmid, pPW416, was confirmed by restriction digest and DNA sequencing. The plasmid was then transformed into *S. elongatus* as previously described [31], and the resulting strain was called PW416. Double DNA recombination was verified by sensitivity to kanamycin, and the strain was segregated by streaking cells on progressively increasing concentrations of erythromycin. The absence of the *hoxYH* genes in the segregated PW416 strain was verified by PCR and southern hybridization.

**Table 1:** Bacterial strains and plasmids used in this study.

*Escherichia coli* strains		
Strain name	Features	Reference
DH10B	F- *mcr*A Δ(*mrr*-*hsd*RMS-*mcr*BC) ϕ80*lac*ZΔM15 Δ*lac*X74 *rec*A1 *end*A1 *ara*D139 Δ (*ara*, *leu*)7697 *gal*U *gal*K λ- *rps*L *nup*G	Invitrogen
*Alteromonas macleodii* “deep ecotype” DSMZ 17117	Wild type	[38]
*Thiocapsa roseopersicina* BBS	Wild type	[39]
*Synechococcus elongatus* strains		
*S. elongatus* PCC 7942	Wild type	Pasteur Culture Collection
PW416	PCC 7942, Δ*hoxYH::*Em^R^	This work
RC41	AltDE hydrogenase operon inserted into NSI site [21] of strain PW416, Em^R^,Sp^R^	This work
Hyn4	*hynShynLhynDhupKhypC1hypC2hypDhypEhypF* from T. roseopersicina in NSII site [21] of strain PW416, Em^R^, Km^R^	This work
Hyn5	*hynShynLhynDhupKhypC1hypC2hypDhypEhypFisp1isp2* from T. roseopersicina in NSII site [21], of strain PW416, Km^R^, Em^R^	This work
Hup	*hupCDHIorf* from *T. roseopersicina* in NSI site of strain PW416, Sp^R^, Em^R^	This work
HoxHup	*hoxEFUYHhupCDHIorf* from *T. roseopersicina* in NSI site of strain PW416, Sp^R^, Em^R^	This work
Wav10	*orf1/cyt/orf2/hynD*/*hupH*/*hypC/hypA/hypB/hypD/hypF/hypE* from AltDE in NSI site of strain PW416, Sp^R^, Km^R^, Em^R^	This work
Hyn4/Wav10	pWav10 construct in NSI site, pHyn4 construct in NSII site of strain PW416, Sp^R^, Km^R^, Em^R^	This work
Hyn5/Wav10	pWav10 construct in NSI site, pHyn5 construct in NSII site of strain PW416, Sp^R^, Km^R^, Em^R^	This work
Hyn4/Hup	pHup construct in NSI site, pHyn4 construct in NSII site of strain PW416, Sp^R^, Km^R^, Em^R^	This work
Hyn5/Hup	pHup construct in NSI site, pHyn5 construct in NSII site of strain PW416, Sp^R^, Km^R^, Em^R^	This work
Hyn5/HoxHup	pHoxHup construct in NSI site, pHyn5 construct in NSII site of strain PW416, Sp^R^, Km^R^, Em^R^	This work
Wav10/HoxHup	pWav10 construct in NSI site, pHoxHup-NSII construct in NSII site of strain PW416, Sp^R^, Km^R^, Em^R^	This work
Plasmids:		
pRL2948a	*mob*, *oriT*, *sacB*, Em^R^Cm^R^	C. P. Wolk, unpublished data
pTRC-NSI	Cloning vector, Sp^R^	[20]
pTRC-NSII	Cloning vector, Km^R^	[20]
pHoxHup-NSI	*T.roseopersicina hoxEFUYH, hupCDHIorf* cloned into pTRC-NSI	This work
pHoxHup-NSII	*T.roseopersicina hoxEFUYH, hupCDHIorf* cloned into pTRC-NSII	This work
pHup-NSI	*T.roseopersicina hoxEFUYH, hupCDHIorf* genes cloned into pTRC-NSI	This work
pHyn4-NSII	*T.roseopersicina hynSL*, *hynD*, *hupK*, *hypC1*, *hypC2*, *hypDEF* genes cloned into pTRC-NSII	This work
pHyn5-NSII	*T.roseopersicina hynSL*, *hynD*, *hupK*, *hypC1*, *hypC2*, *hypDEF*, *isp1*, *isp2* genes cloned into pTRC-NSII	This work
pWav10	*orf1/cyt/orf2/hynD*/*hupH*/*hypC/hypA/hypB/hypD/hypF/hypE* inserted into NSI site, Δ*hynSL*, cloned into pTRC-NSI, Sp^R^	[16]
pPW416	*S. elongatus* hox region (Δ*hoxYH::*Em^R^) cloned in pUC19, Em^R^, Cm^R^	This work
pRC41	*orf1/cyt/orf2/hynD*/*hupH*/*hynS/hynL*/*hypC/hypA/hypB/hypD/hypF/hypE* inserted into NSI site, cloned into pTRC-NSI, Sp^R^	[16]

**Table 2:** PCR primers used in this study.

Primer Name	Sequence (5′ – 3′)
Isp-F	TATCAATTTAAATTGGCCAAGAAGCAGACCAAG
Isp-R	GAGGCGCGCCTCATGTCAGTTCTTCTCCAC
HupCDHIorf-F	TTAATCTCATATGCGGACCTGGCGGGGA
HupCDHIorf-R	TCACTAGTGAGAGAAATTCCACTCCGG
TrHox-F1	ACAGACCATGGAATTCGAGCTCAAGGAGGAATAACATATGAGTCTGCAGCAAGCCAAGCC
TrHox-R1	AATAATCACCTGAAACGCGTCCCCGCCAGGTCCGCATATGTTATTCCTCCCTTCAGCCGCGCCTGAGTGTGTCG
EmCm-F	TTATACTCGAGCACGTTCCATGGCCTCCAT
EmCm-R	AATATACTAGTCTGTCATGCCATCCGTAAGATGC
Hox11	AATCGAAGCTTCGAGTCCATAGCGATGGC
Hox12	TTATTGGATCCAACAGGACTGAAGACGACCTC
Hox15	TTAATCTCGAGATTGCGGAGATGGTTGAAGAC
Hox16	TAATTTCTAGAAATTGAGCAGGCACTGACTC

The plasmid pHyn4 was previously described [16]. To make pHyn4-NSII, the entire cluster of *T. roseopersicina* genes in pHyn4 was digested with NdeI and AscI and ligated into a similarly digested pTRC-NSII. To make pHyn5-NSII, the *isp1isp2* genes were amplified by PCR from *T. roseopersicina* genomic DNA using primers Isp-F and Isp-R, digested with SwaI and AscI, and ligated into SwaI and AscI-digested pHyn4-NSII. The resulting plasmid, pHyn5-NSII, contains the *isp1isp2* genes downstream of the cluster including the *hynSL*, *hynD*, *hupK*, and *hypC1C2DEF* genes.

To make pHup-NSI, the genes encoding *hupCDHI* and an additional open reading frame (*orf*) of unknown function that is located after *hupI* [18] were amplified by PCR from *T. roseopersicina* genomic DNA using primers HupCDHIorf-F and HupCDHIorf-R. The resulting PCR product was digested with NdeI and SpeI and ligated into an NdeI/SpeI-digested pTRC-NSI. To make pHoxHup-NSI, the *hoxEFUYH* [17] genes were amplified from *T. roseopersicina* genomic DNA using primers TrHox-F1 and TrHox-R1. These primers were designed so that each end of the resulting product contained 40-bp of sequence homology to the pHup-NSI vector. The plasmid, pHup-NSI was digested with EcoRI and NdeI and assembled with the *hoxEFUYH* PCR product using the one-step isothermal “chewback and anneal” (CBA) assembly method [37]. The resulting plasmid was called pHoxHup-NSI. To make pHoxHup-NSII, the entire cluster of *T. roseopersicina* genes was digested with NcoI and SpeI, and ligated into an NcoI/SpeI-digested pTRC-NSII vector.

### Hydrogenase activity assays

*In vitro* hydrogen evolution assays were performed as described in [28] with the following modifications. Cells (500 ml) were centrifuged, resuspended in 1 ml sonication buffer (10 mM Tris-HCl, pH 7, 0.5 mM EDTA, 1 mM DTT), and sonicated (under aerobic conditions) two times for 2 minutes each on ice and centrifuged to remove cell debris before being used for assays. Reactions were performed under anaerobic conditions at 30°C as described previously using the chemical electron donor, methyl viologen [28].

### Protein techniques

SDS-polyacrylamide gel electrophoresis (SDS-PAGE) was performed according to [34]. Gels were either stained with Coomassie using the SimplyBlue SafeStain reagent (Invitrogen) or transferred to nitrocellulose for Western blotting using polyclonal rabbit antibodies specific for *T. roseopersicina* HynL and HynS as the primary antibodies [28], [34].

## Supporting Information

Figure S1 **Southern blot confirmation of the** ***S. elongatus hoxYH*** **mutant (PW416).** After segregation on increasing antibiotic concentration, chromosomal DNA was digested with EcoRI and HindIII for Southern blotting from the following samples: Lane 1) Wild-type, 2) PW416-1, 3) PW416-2, and 4) PW416-3. **A.** Southern blot hybridized with a labeled PCR product amplified from *hoxU*. **B.** Southern blot hybridized with a labeled PCR product amplified from *hoxYH*. **C.** Restriction map of the wild-type *S. elongatus hoxYH* region. **D.** Restriction map of the PW416 mutant *hoxYH* region.
(TIF)

Figure S2 **PCR confirmation of the** ***S. elongatus hoxYH*** **mutant (PW416).** After segregation on increasing antibiotic concentration, chromosomal DNA was isolated and used for PCR. The templates used in each lane are the following: Lane 1) Wild-type, 2) PW416-1, 3) PW416-2, 4) PW416-3, 5) no template, 6) pPW416 plasmid DNA, and 7) *S. elongatus* PCC 7942 chromosomal DNA. **A.** PCR products amplifying *hoxH* using primers Hox23 and Hox24 (Table S1). **B.** Diagram of primer binding sites in *S. elongatus* **C.** PCR products amplifying *hoxU* through *hoxW* using primers Hox16 and Hox17 (Table S1). **D.** Diagram of primer binding sites in wild-type *S. elongatus hoxYH* region. **E.** Diagram of primer binding sites in PW416 *hox* mutant.
(TIF)

Table S1 Primers used in the Supplemental Figures.
(TIF)

Table S2 *In vitro* hydrogen evolution activity assay on wild-type and segregated *hoxYH* mutant strains.
(TIF)
