# Supplementary figures and images for: Heterologous Expression of Alteromonas macleodii and Thiocapsa roseopersicina [NiFe] Hydrogenases in Synechococcus elongatus
Source: PLoS One. 2011 May 26;6(5):e20126. doi: 10.1371/journal.pone.0020126 (PMC3102683; doi:10.1371/journal.pone.0020126)

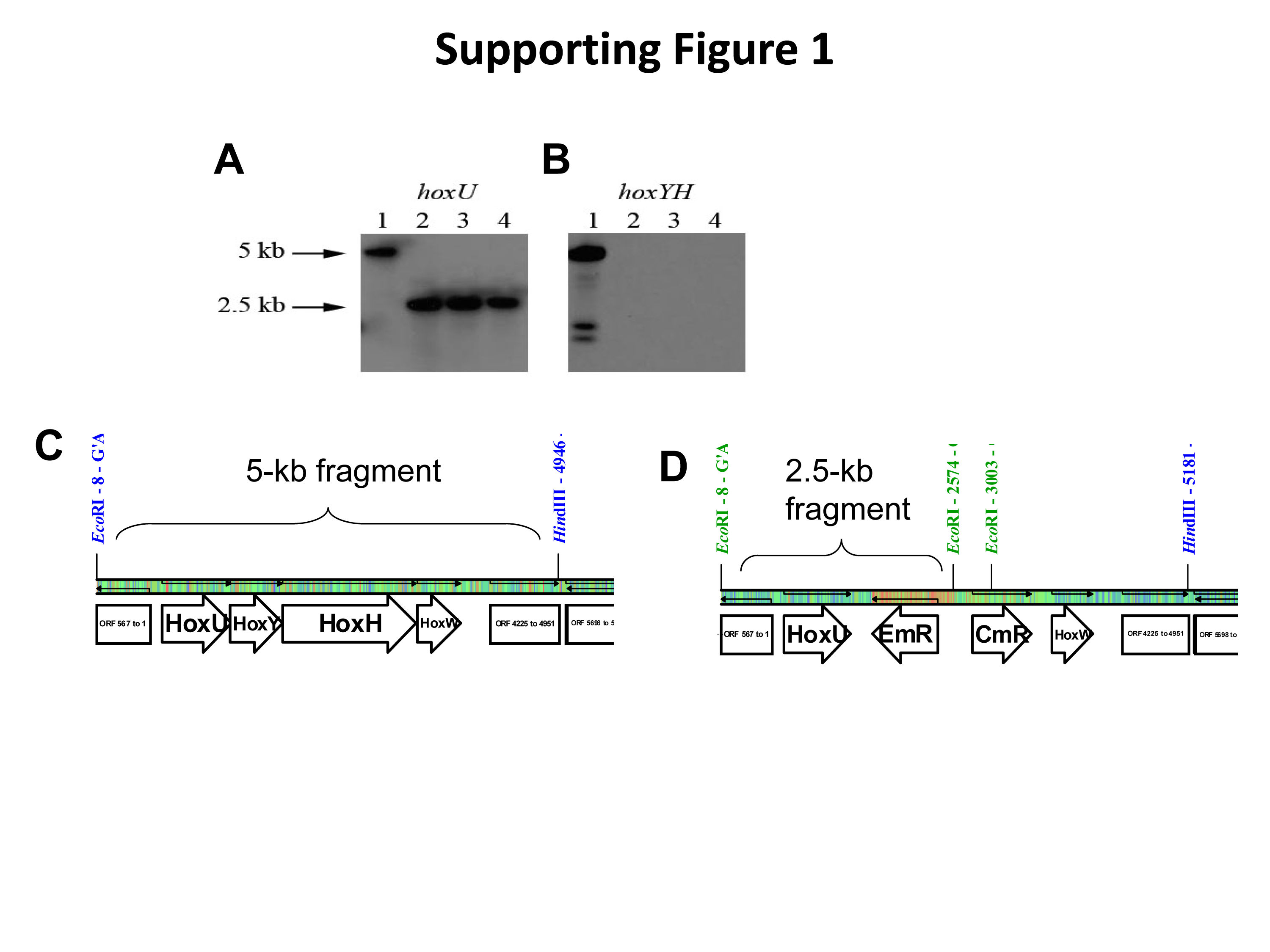

Supplement: Figure S1 — Southern blot confirmation of the S. elongatus hoxYH mutant (PW416). After segregation on increasing antibiotic concentration, chromosomal DNA was digested with EcoRI and HindIII for Southern blotting from the following samples: Lane 1) Wild-type, 2) PW416-1, 3) PW416-2, and 4) PW416-3. A. Southern blot hybridized with a labeled PCR product amplified from hoxU. B. Southern blot hybridized with a labeled PCR product amplified from hoxYH. C. Restriction map of the wild-type S. elongatus hoxYH region. D. Restriction map of the PW416 mutant hoxYH region. (TIF) [file pone.0020126.s001.tif]

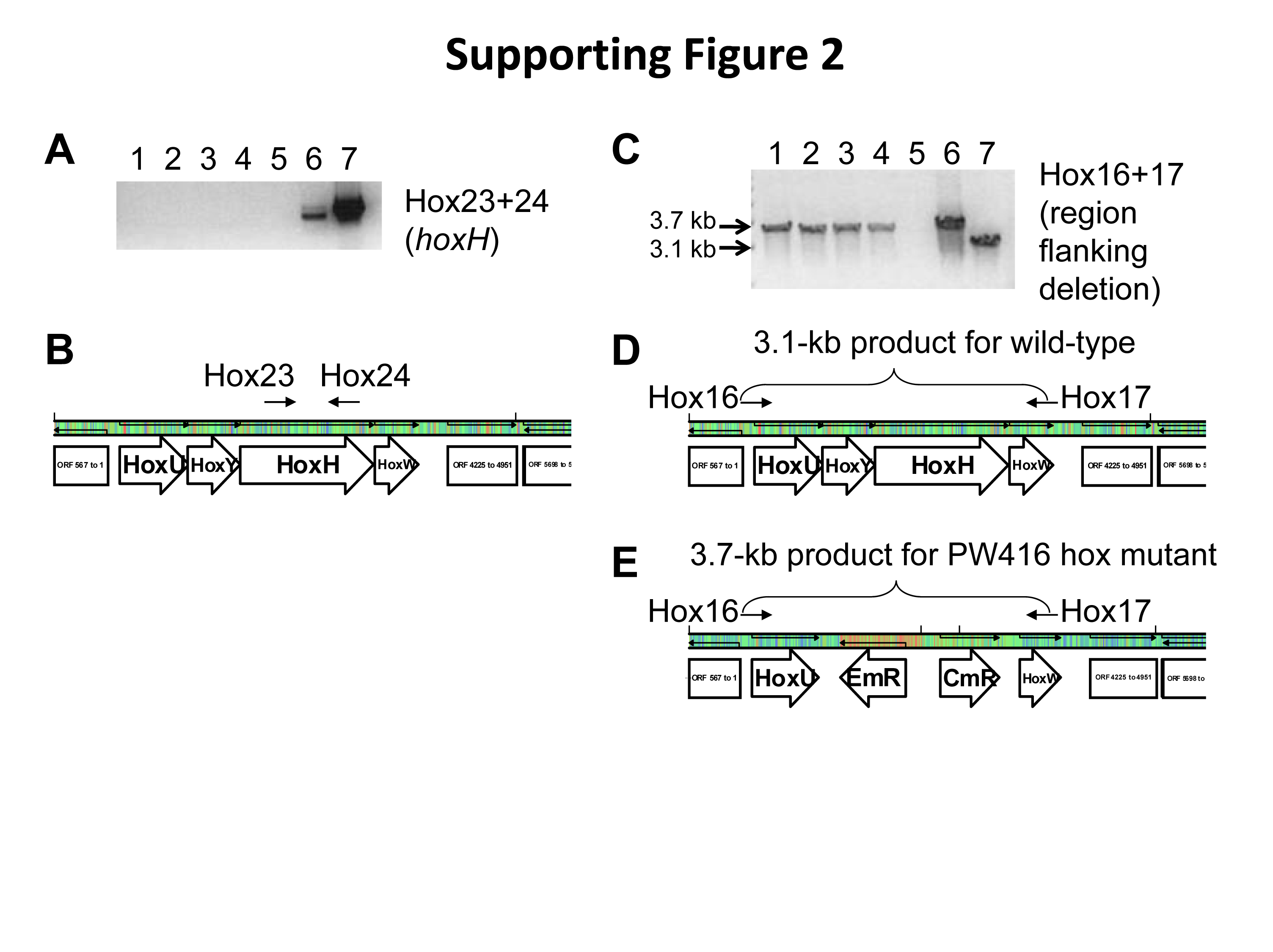

Supplement: Figure S2 — PCR confirmation of the S. elongatus hoxYH mutant (PW416). After segregation on increasing antibiotic concentration, chromosomal DNA was isolated and used for PCR. The templates used in each lane are the following: Lane 1) Wild-type, 2) PW416-1, 3) PW416-2, 4) PW416-3, 5) no template, 6) pPW416 plasmid DNA, and 7) S. elongatus PCC 7942 chromosomal DNA. A. PCR products amplifying hoxH using primers Hox23 and Hox24 (Table S1). B. Diagram of primer binding sites in S. elongatus C. PCR products amplifying hoxU through hoxW using primers Hox16 and Hox17 (Table S1). D. Diagram of primer binding sites in wild-type S. elongatus hoxYH region. E. Diagram of primer binding sites in PW416 hox mutant. (TIF) [file pone.0020126.s002.tif]

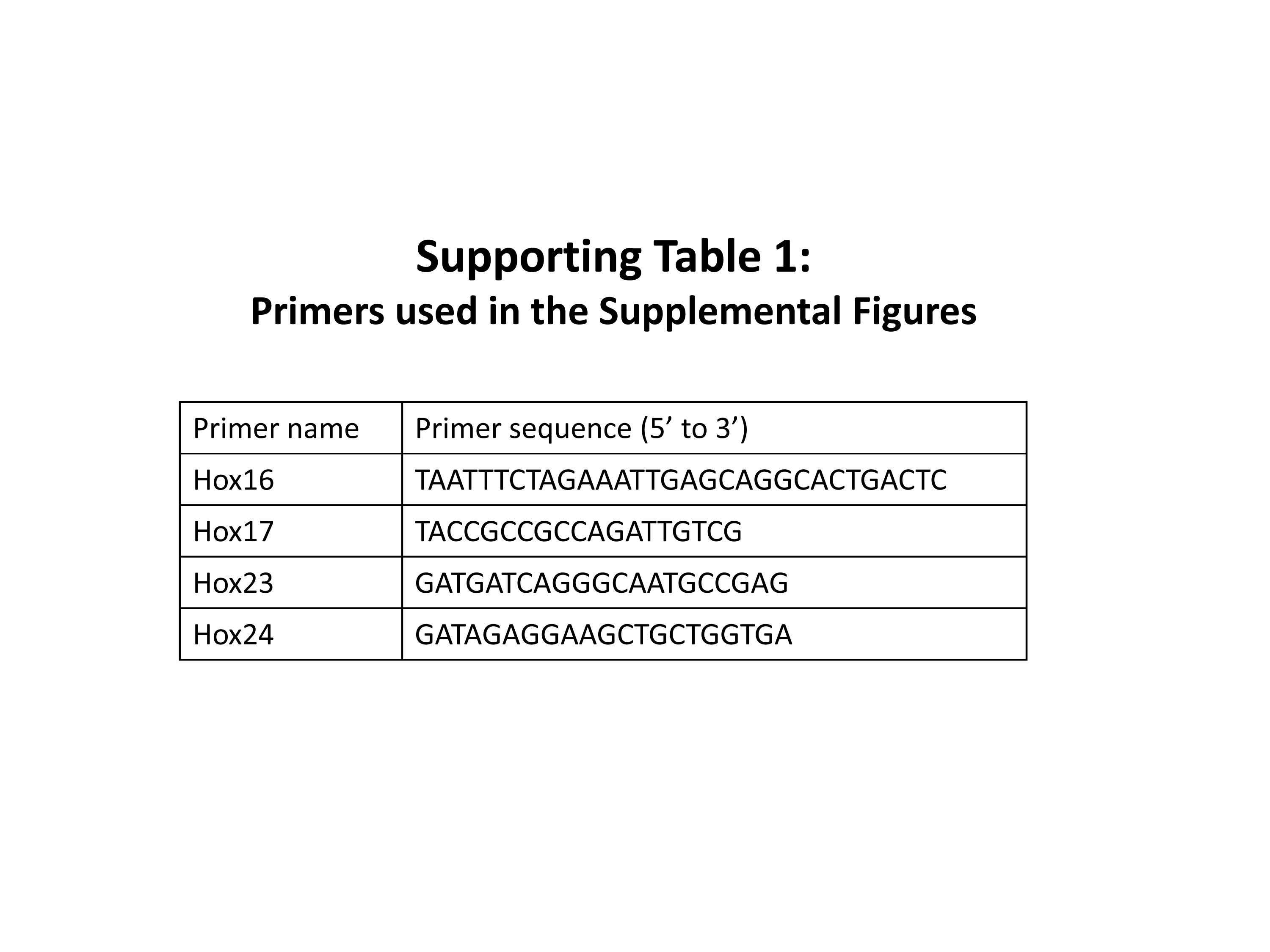

Supplement: Table S1 — Primers used in the Supplemental Figures. (TIF) [file pone.0020126.s003.tif]

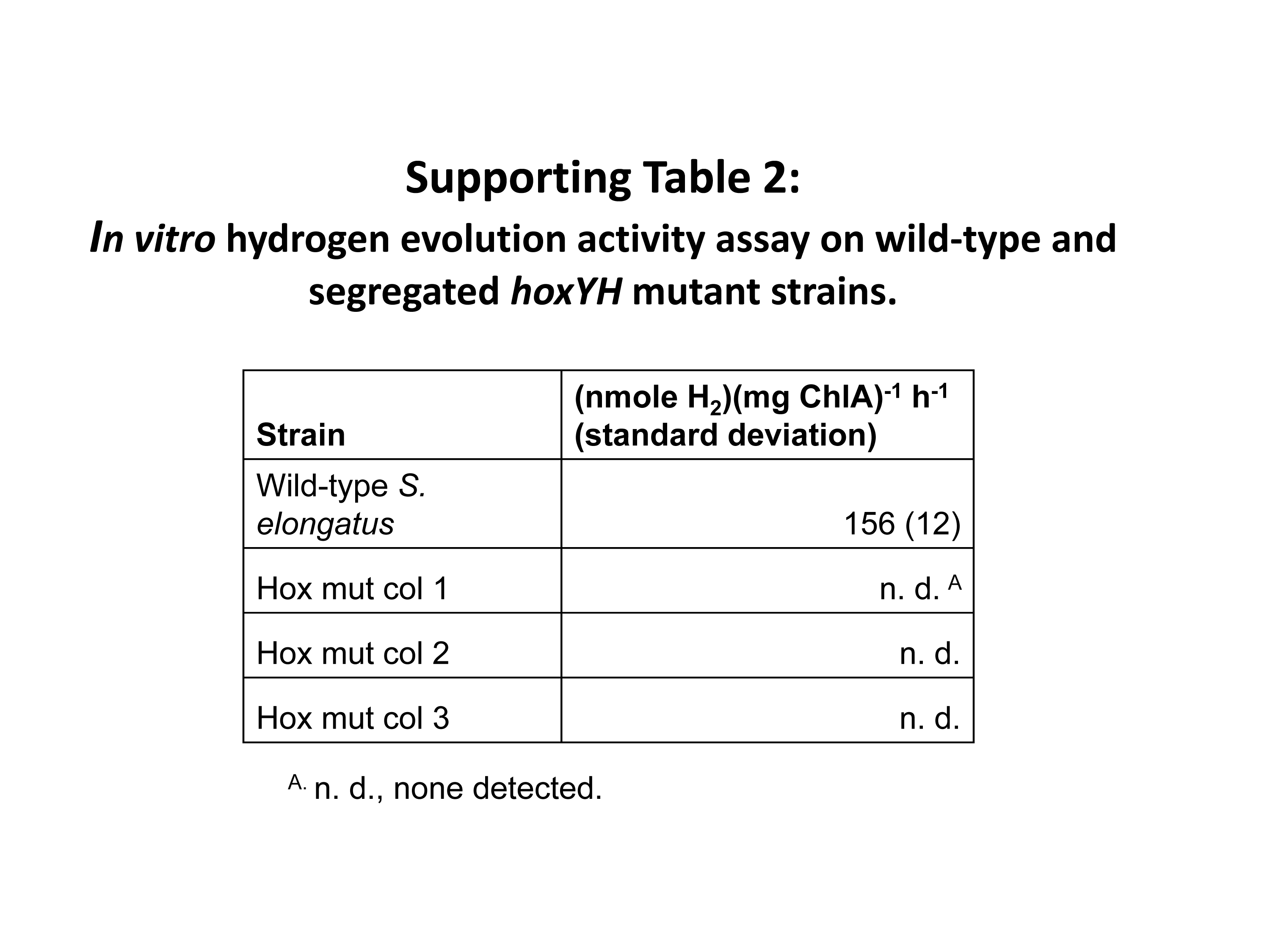

Supplement: Table S2 — In vitro hydrogen evolution activity assay on wild-type and segregated hoxYH mutant strains. (TIF) [file pone.0020126.s004.tif]
